# Supplementary figures and images for: Neurons Expressing Pathological Tau Protein Trigger Dramatic Changes in Microglial Morphology and Dynamics
Source: Front Neurosci. 2019 Nov 7;13:1199. doi: 10.3389/fnins.2019.01199 (PMC6855094; doi:10.3389/fnins.2019.01199)

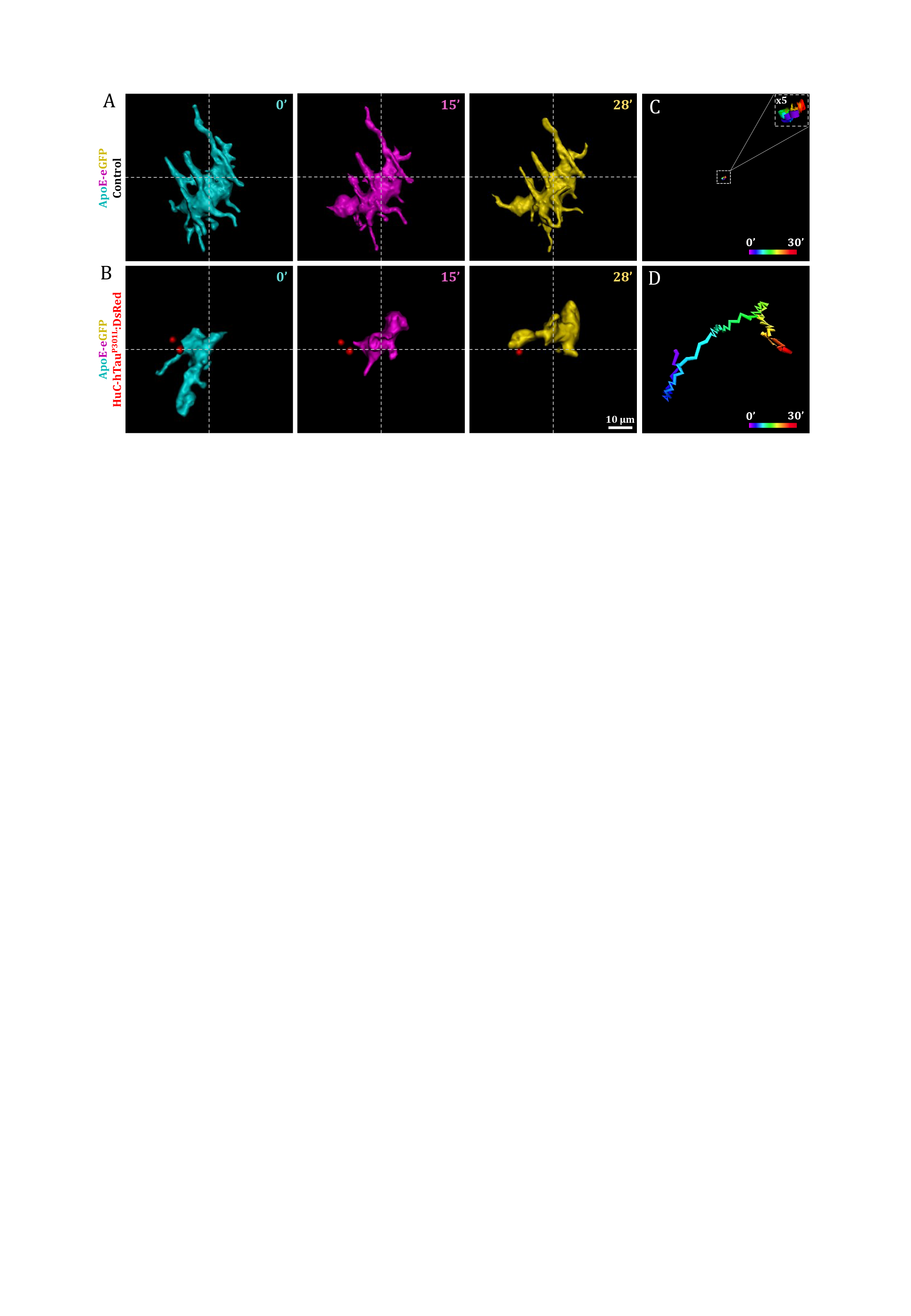

Supplement: Supplementary file 9 [file Image_1.TIF]

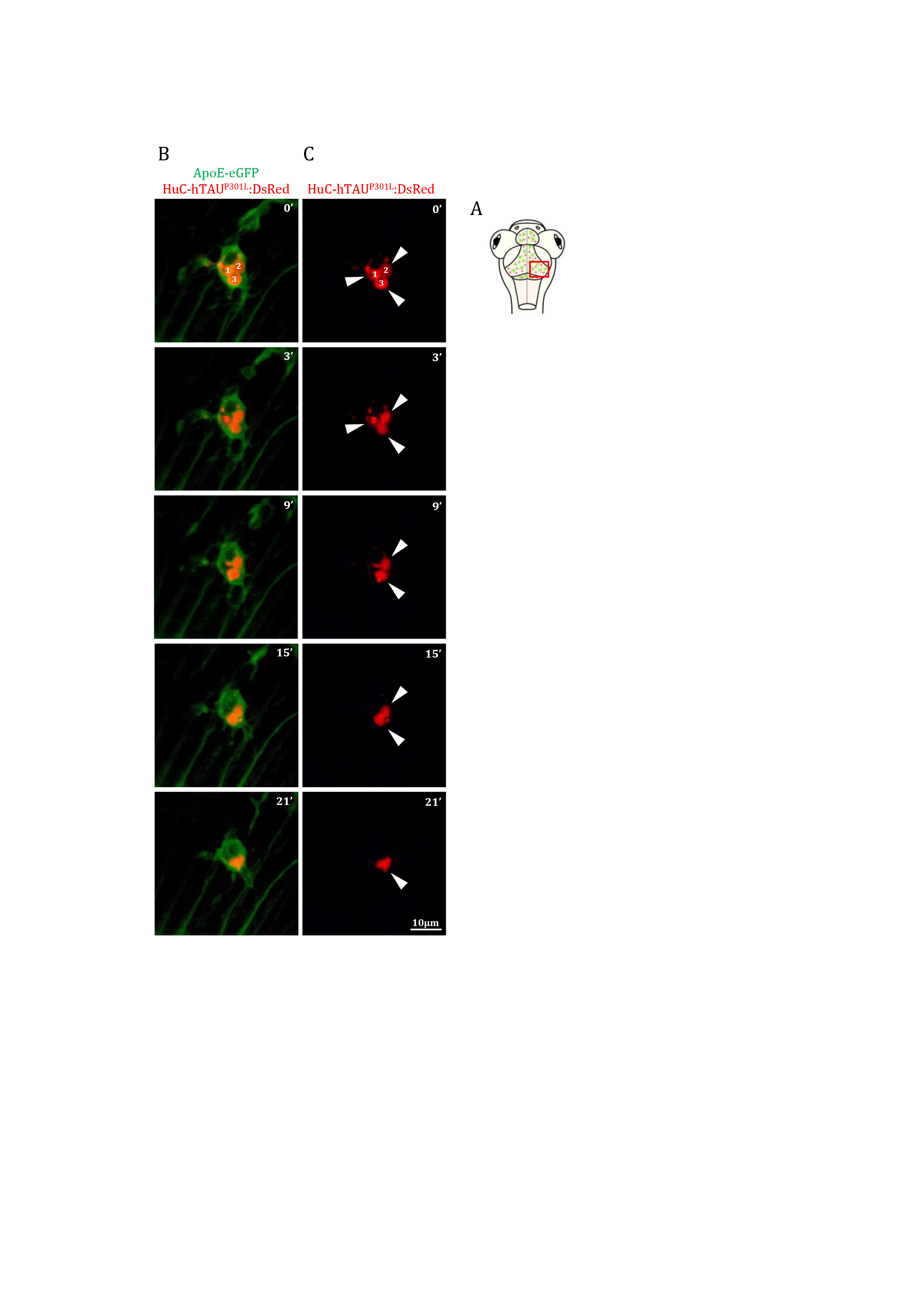

Supplement: Supplementary file 10 [file Image_2.TIF]
